# Metastatic Adenocarcinoma of Intestinal Origin in Reconstructed Ureters

**DOI:** 10.7759/cureus.55513

**Published:** 2024-03-04

**Authors:** Priyanka Venkatesh, Weijing Sun, Louis Wetzel, Anup Kasi

**Affiliations:** 1 Department of Internal Medicine, University of Kansas Medical Center, Kansas City, USA; 2 Department of Medical Oncology, University of Kansas Medical Center, Kansas City, USA; 3 Department of Radiology, University of Kansas Medical Center, Kansas City, USA

**Keywords:** chemotherapy, metastatic, reconstructed ureter, eagle-barrett, adenocarcinoma

## Abstract

In patients with long ureteral defects, the use of bowel segments for reconstruction is an effective but suboptimal alternative because the bowel is not resistant to the potential carcinogenic effects of urine. Primary malignancies in reconstructed conduits have been scarcely described in the literature. This case report elaborates on a case of metastatic adenocarcinoma arising in ureters reconstructed using small intestinal segments. A 49-year-old with Eagle-Barrett syndrome presented with abdominal pain and was found to have irregular enhancement of the right ureteropelvic junction and small, non-specific liver lesions. Biopsy of the liver lesions showed poorly differentiated adenocarcinoma with immunohistochemistry staining consistent with small intestinal origin. The patient was treated as a tumor of GI origin with chemotherapy and subsequently underwent microwave ablation of his liver metastases. He also received concurrent chemoradiation for residual disease at the ureteral conduit. PET scan images done after completion of treatment showed resolution of all lesions. Further research into alternative structures that could be used to create conduits and screening methods for these patients is imperative to reduce the incidence of such malignancies.

## Introduction

The ureters are essential for maintaining normal renal function by providing low pressure for urinary drainage and avoiding urinary reflux and stasis. Repair of long ureteral defects has remained a challenge for urologists because of the complications associated with intestinal substitution [1]. This approach, however, is suboptimal because intestinal segments, unlike the urothelium, are not resistant to the inflammatory and potential carcinogenic effects of urine. Ileal segments are most commonly used, followed by appendiceal and colonic segments. The long-term exposure of these segments to the toxic effects of urine leads to various complications, which has necessitated constant evolution in these techniques [2]. Primary malignancies in reconstructed conduits have been scarcely described in the literature. This case report elaborates on a case of metastatic adenocarcinoma arising in ureters reconstructed using small intestinal segments.

## Case presentation

**Figure 1:**
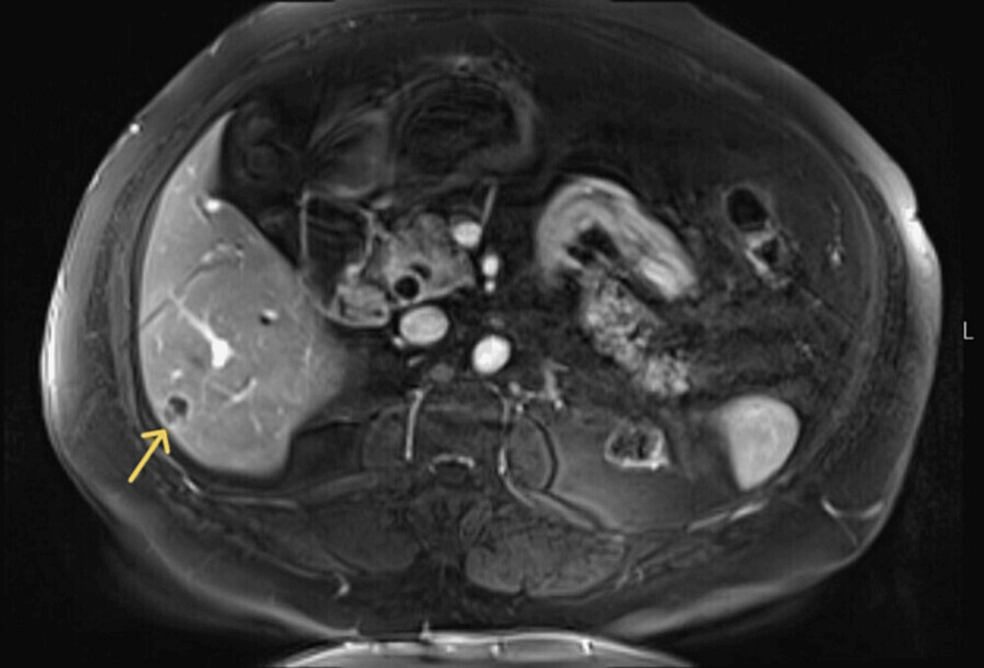
Axial post-contrast fat-suppressed T1-weighted image shows a small segment 6 liver lesion (arrow) with low signal centrally and peripheral enhancement

**Figure 2:**
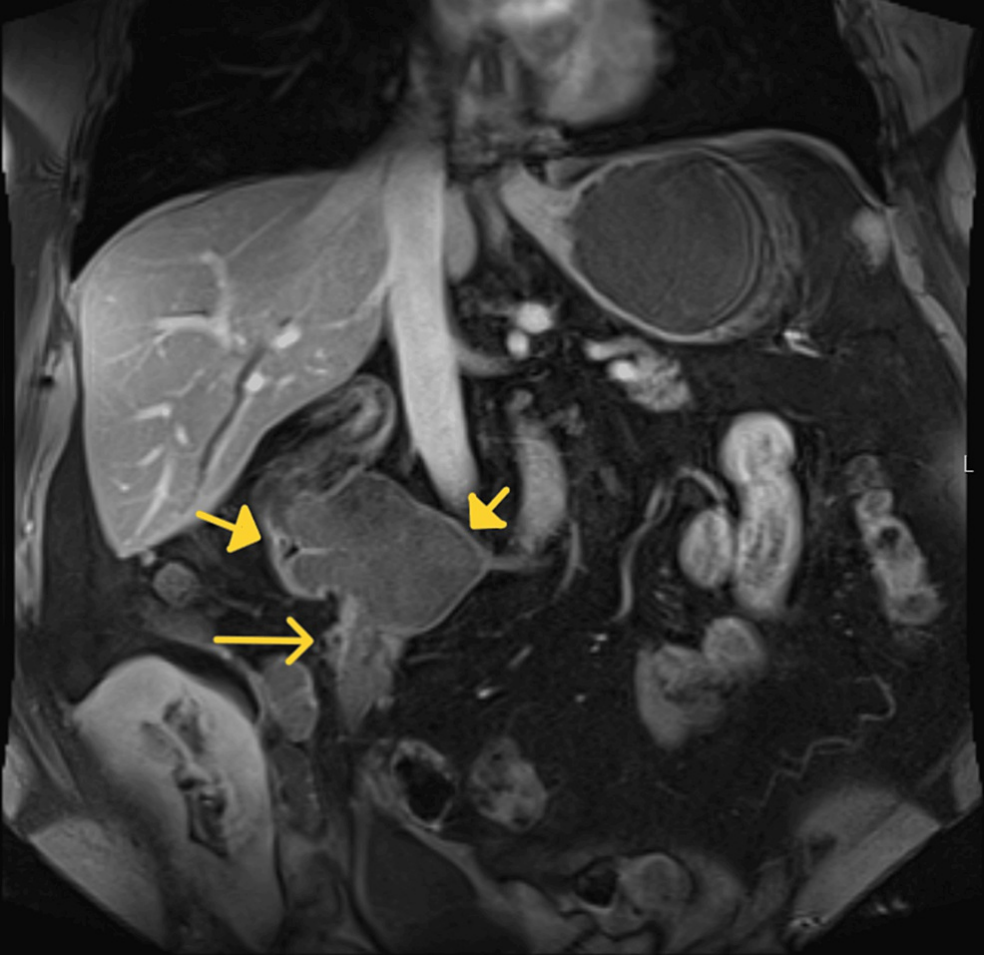
Coronal post-contrast fat-suppressed T1-weighted image shows a dilated right renal pelvis (solid arrows) and questionable linear enhancement along the lateral aspect of the right ureteropelvic junction (arrow)

**Figure 3:**
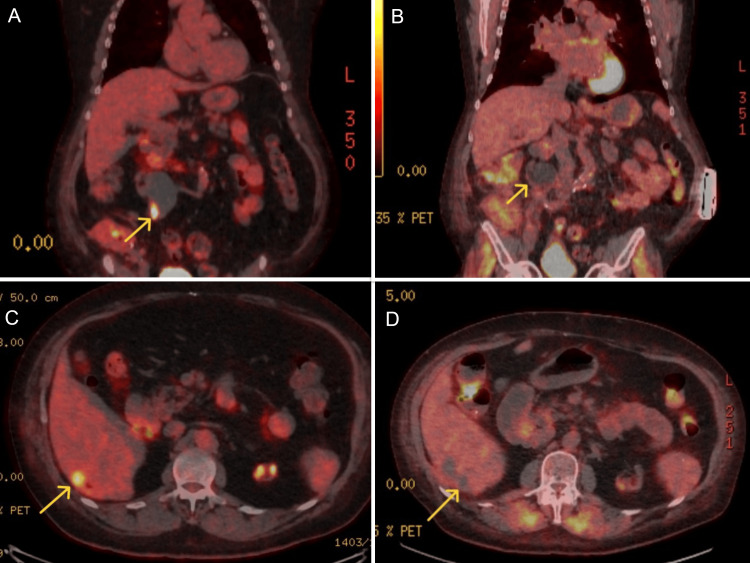
Coronal PET images showing an area of hypermetabolic activity in the lateral aspect of the dilated right ureter (A) and resolution on post-treatment images (B) done after completion of therapy. Similarly, note the area of hypermetabolic activity at the site of liver metastasis on axial PET image (C) and subsequent resolution (D). PET: positron emission tomography

**Figure 4:**
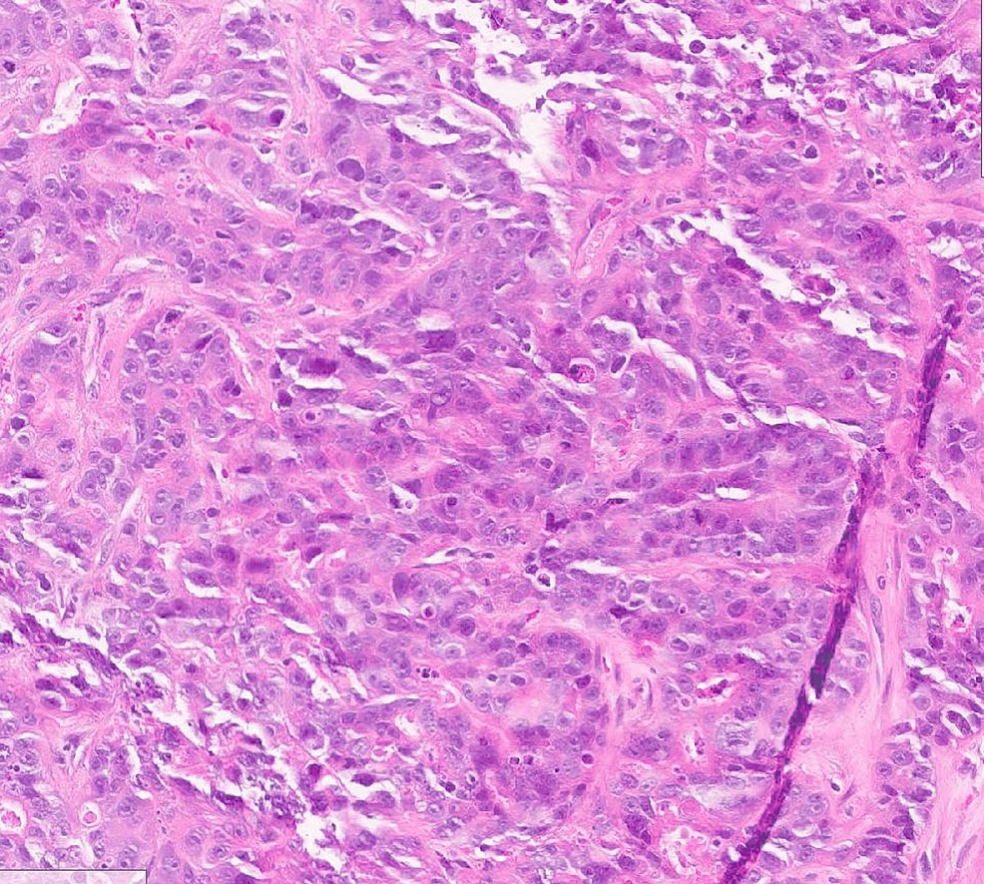
H&E stain, 40x magnification; showing poorly differentiated adenocarcinoma H&E: hematoxylin and eosin

**Figure 5:**
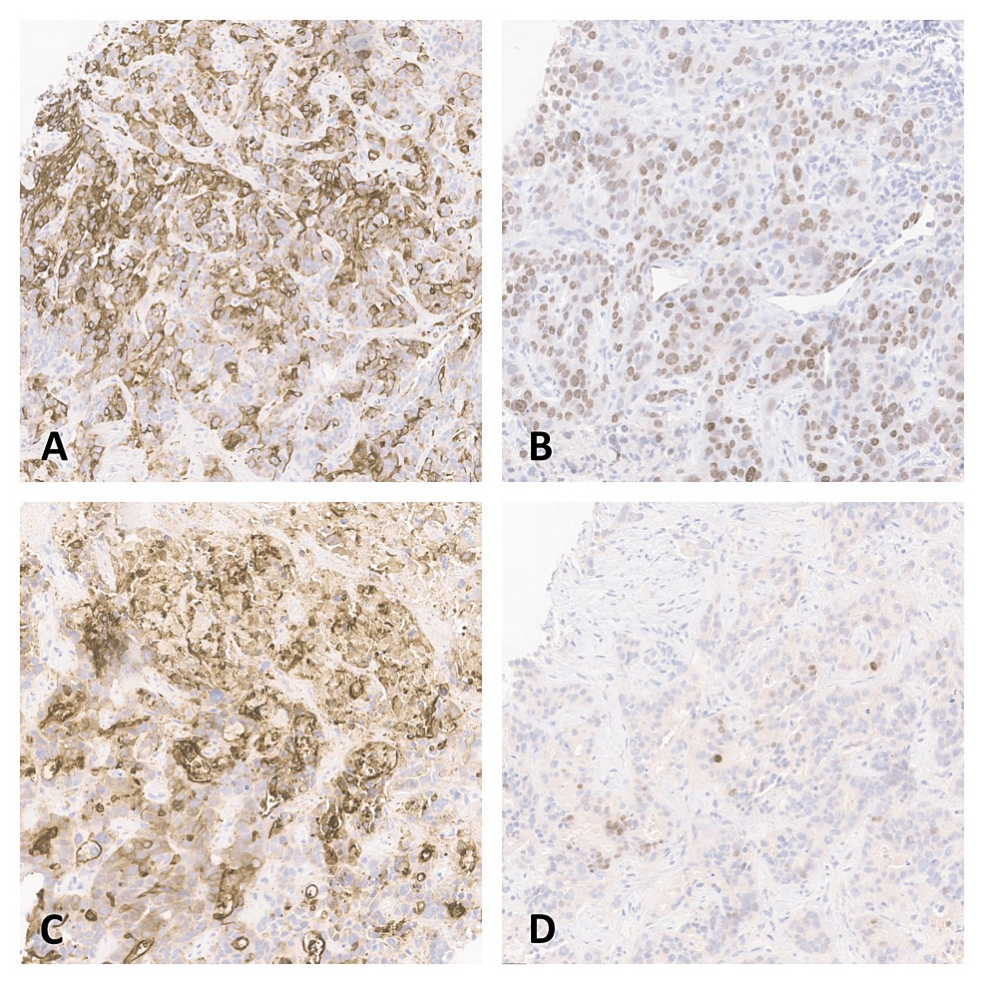
Immunohistochemistry staining of liver biopsy specimen demonstrating positive CK20 (A), positive SATB2 (B), positive Villin (C), and positive CDX2 (D)

A 49-year-old patient with a past medical history significant for Eagle-Barrett syndrome, end-stage renal disease (ESRD) s/p cadaveric renal transplant, hypertension, and chronic obstructive pulmonary disease presented to an outside facility with abdominal pain, constipation, and intermittent hematochezia. Vital signs and basic laboratory investigations were unremarkable on admission. On evaluation with computed tomography (CT) of the abdomen and pelvis, the patient was found to have a mass in the right renal pelvis and multiple lesions in the liver. Magnetic resonance imaging (MRI) of the abdomen and pelvis was performed, which showed segment 6 and 7 lesions in the liver with features of complex fluid content and an increase in the size of the previously noted right renal mass, which was found to contain complex hemorrhage/debris (Figure 1 and Figure 2). A positron emission tomography (PET) scan obtained showed the presence of a hypermetabolic periureteric nodule suspicious for primary malignancy and focal hypermetabolism along the lateral right ureteropelvic junction (Figure 3A). There were also three hypermetabolic hepatic metastases (Figure 3C). Urology performed a cystourethroscopy with ureteroscopy but was unable to cannulate the right ureteral orifice and therefore could not obtain biopsies of the hypermetabolic lesions in the ureter. Interventional radiology (IR) was consulted to attempt a biopsy of the right renal mass. As the liver lesions were more amenable to biopsy or aspiration, IR performed a CT-guided biopsy of the peripheral liver lesions, which was positive for poorly differentiated adenocarcinoma with necrosis (Figure 4). Staining was positive for CK20, SATB2, Villin, and CDX2 (Figure 5) and negative for CK7, Napsin, TTF1, GATA3, PAX-8, and NKX 3.1. The immunohistochemical profile was consistent with colorectal or small bowel adenocarcinoma. Tissue was sent for genomic profiling and there were no actionable mutations found. To further clarify the diagnosis, multiple tumor markers were checked, including AFP, CEA, PSA, Beta-hCG, and LDH. CEA was elevated at 13.8 ng/mL (reference range: < 3 ng/mL), which further confirmed the suspicion of GI malignancy. A colonoscopy was performed, which showed the presence of a polyp in the cecum but no masses suspicious of malignancy. The biopsy of the polyp was consistent with tubular adenoma.

Clinical course and treatment

The patient was treated as a tumor of GI origin with metastasis to the liver. The patient completed 10 cycles of FOLFOX (folinic acid, fluorouracil, and oxaliplatin), which includes 5-FU and oxaliplatin. After cycle 10, oxaliplatin was discontinued due to neuropathy for the last 3 cycles. After 1 cycle of chemotherapy, CEA reduced to 6.1 ng/mL from 13.8 ng/mL. His treatment course was complicated by uncontrolled nausea and vomiting and coronavirus disease 2019 (COVID-19) infection requiring hospitalizations. Repeat imaging showed a decrease in the size of liver metastases and ureteral mass. Due to the presence of persistent metastases in the liver and mass in the ureter, the patient was discussed at a multidisciplinary tumor board. The patient underwent microwave ablation (MVA) to the liver, as he was not a surgical candidate. The patient was then evaluated by radiation oncology for residual disease at the ureteral conduit and received concurrent chemoradiation. CT simulation was done and two planning target volumes (PTV) were identified - a dose of 5500 centigray (cGy) in 25 fractions (Fr) of 220 cGy was delivered to the first target volume and a dose of 5000 cGy in 25 Fr of 200 cGy was delivered to the second target volume with 6 MV photons using intensity-modulated radiation therapy (volumetric modulated arc radiotherapy technique) utilizing a simultaneous integrated boost. Capecitabine was started on days of radiation - 1500 mg in the mornings and 2000 mg in the evenings. A repeat PET scan done after completion showed a resolution of hypermetabolic hepatic metastases and no new suspicious lesions. There was also resolution of the hypermetabolic focus in the right ureteropelvic junction (Figure 3C and Figure 3D).

## Discussion

Eagle-Barrett syndrome also known as Prune-Belly syndrome is a rare condition that presents with characteristic urological abnormalities [3]. Our patient was born with bilateral ureteral defects and subsequently underwent surgery involving the transposition of loops of the small intestine to repair the defects. As noted on imaging, prior surgical reconstruction involved anastomosis of the left ureter to the right renal pelvis and right ureteral reimplantation into the bladder dome with cephalic tethering of the bladder dome.

In our patient, prior clinical records indicate that the patient’s ureters were reconstructed from the small bowel. Immunohistochemistry (IHC) staining was positive for CK20, CDX2, SATB2, and Villin and negative for CK7, consistent with adenocarcinoma of intestinal origin. Small bowel adenocarcinoma stains were positive for CK20, CDX-2, and Villin [4]. In a case series published by Neri et al. reporting the immunophenotypic profiles of 100 cases of small bowel adenocarcinoma, the CK20+/CK7- staining profile was seen in 50% of their cases, 67% of cases were positive for CDX2, and SATB2 positivity was seen in 20% of the cases [5]. In another case series of 30 patients with small intestinal adenocarcinomas by Zhang et al., 66% showed Villin positivity [6]. Although this staining pattern is also seen in colorectal adenocarcinoma, our patient's clinical presentation and prior surgical history make small bowel adenocarcinoma the most likely diagnosis. The patient was treated as adenocarcinoma of intestinal origin with FOLFOX.

Due to the persistent tumor burden after 13 cycles of chemotherapy, the consensus from the tumor board was to consider surgery for the liver lesions among other options such as external beam radiation, microwave ablation, radiofrequency ablation, trans-arterial chemoembolization, cryoablation, and others. The patient was not a surgical candidate and hence, microwave ablation of the liver lesions was successfully completed by interventional radiology. Microwave ablation of liver metastases is currently reserved for patients who are not good candidates for surgical resection or for those who have failed other therapy options [7,8]. The evidence for MWA is mostly retrospective and generally targeted toward colorectal metastases. Because of the ability to create higher temperatures without limitation from charring and vaporization, MWA is gaining popularity over other modalities. For the patient’s right ureteral mass, the initial plan was to attempt stereotactic body radiation therapy. However, this required fiducial placement for tumor localization. Ultimately, it was determined that standard radiation was the safer option for this patient. Fiducials are generally used to provide additional guidance for stereotactic radiosurgery by optimizing the linear geometry at the tumor border. They improve the spatial visualization of the tumor margins, which helps improve dosing strategies and better focus stereotactic radiosurgery to the tumor and avoid healthy tissue [9].

The use of intestinal segments for the repair of long ureteral defects has continued to remain a challenge for urologists because of the complexity of the procedure and potential complications. Contact with urine has been implicated in carcinogenesis through anastomotic site inflammation, formation of nitrosamines, and epithelial hyperplasia [10]. In prior literature, carcinomas arising in the ureterosigmoidostomies have been frequently described. A 100-fold increased risk of malignancy was described, with a latency of 20-30 years [11,12]. With this background, most urinary diversion procedures adopt the use of ileal conduits instead, as malignancy of the small intestine is very rare. Ileal segments are most commonly used followed by appendiceal interposition and reconfigured colonic substitution. The first case of ureteral reconstruction using a small bowel was described by Shoemaker in 1911 [13]. Four cases of malignant neoplasms of small intestinal origin in reconstructed ureters have been described in the literature to our knowledge. Matos et al. described a case of adenocarcinoma in a 79-year-old woman arising from an ileal ureter used for reconstructive surgery at the age of 38 [2]. Another case of adenocarcinoma of the ileal segment was described by Trzepizur et al. in a 51-year-old female patient who underwent surgery in childhood for bladder extrophy [14]. Kobayashi et al. reported a case of adenocarcinoma of the small bowel in a reconstructed ureter in a 65-year-old man who underwent surgery for a tuberculous ureteral stricture 45 years prior [15]. England and Salter described ileal-ureter adenocarcinoma in a patient who underwent surgical treatment for vesicoureteric reflux in early adolescence [10]. Similar to our patient, their patient developed ESRD after reconstruction and received a kidney transplant.

## Conclusions

The development of adenocarcinomas in ileal conduits has gained recognition due to previous reports in the literature. This knowledge acquired from prior literature can effectively guide workups in patients with a history of reconstructed anatomical conduits of small intestinal or colorectal origin. We would like to emphasize the need to have a high index of suspicion for intestinal malignancy in such cases. Methods of effective screening in such patients are not clearly elucidated and need further studies. Research into reconstruction using urothelium-lined structures for the creation of conduits that would be exposed to urine may have the potential to reduce the incidence of such cancers.
